# Time to Command-Following and Outcomes After Traumatic Brain Injury

**DOI:** 10.1001/jamanetworkopen.2024.49928

**Published:** 2024-12-10

**Authors:** Samuel B. Snider, Hansen Deng, Flora M. Hammond, Robert G. Kowalski, William C. Walker, Ross D. Zafonte, David O. Okonkwo, Joseph T. Giacino, Ava M. Puccio, Yelena G. Bodien

**Affiliations:** 1 Division of Neurocritical Care, Department of Neurology, Brigham and Women’s Hospital, Harvard Medical School, Boston, Massachusetts; 2 Department of Neurological Surgery, University of Pittsburgh Medical Center, Pittsburgh, Pennsylvania; 3 Department of Physical Medicine and Rehabilitation, Indiana University School of Medicine, Indianapolis; 4 Departments of Neurosurgery and Neurology, University of Colorado School of Medicine, Aurora; 5 Department of Physical Medicine and Rehabilitation, School of Medicine, Virginia Commonwealth University, Richmond; 6 Department of Physical Medicine and Rehabilitation, Harvard Medical School, Boston, Massachusetts; 7 Department of Physical Medicine and Rehabilitation, Spaulding Rehabilitation Hospital, Charlestown, Massachusetts; 8 Department of Neurosurgery, Neurotrauma Clinical Trials Center, University of Pittsburgh Medical Center, Pittsburgh, Pennsylvania; 9 Center for Neurotechnology and Neurorecovery and Department of Neurology, Massachusetts General Hospital, Boston

## Abstract

This cohort study examines the association of time to command-following with death or dependency at 1 year among individuals with moderate-severe traumatic brain injury (TBI).

## Introduction

After a traumatic brain injury (TBI), the persistent absence of command-following (ie, behavioral responses to verbal instructions) is often considered to be an indicator of poor prognosis.^1,2,3^ However, the accuracy of prognoses based on the absence of command-following at specific postinjury time points is unclear.

We measured the association between time to command-following and 1-year outcomes in more than 9000 participants enrolled in 2 large prospective TBI studies, the TBI Model Systems (TBIMS) National Database^4^ and the University of Pittsburgh Brain Trauma Research Center (BTRC) Database.^5^

## Methods

### Study Cohorts

This cohort study was approved by institutional review boards at all sites, and participants’ surrogates provided informed consent. This report follows the Strengthening the Reporting of Observational Studies in Epidemiology (STROBE) reporting guideline for cohort studies. TBIMS^6^ includes participants aged at least 16 years with moderate-severe TBI admitted to 1 of 16 US inpatient rehabilitation hospitals. The BTRC database^5^ includes participants aged 16 to 80 years with severe TBI (admission Glasgow Coma Scale [GCS] score ≤8 with motor GCS ≤5) admitted to 1 US level-I trauma center, excluding those with GCS of 3 and bilaterally fixed and dilated pupils. TBIMS collected self-reported race and ethnicity data, categorized as Asian or Pacific Islander, Black, Hispanic, White, or other (ie, people who did not self-report into any of these categories). BTRC collected self-reported race data as Asian or Pacific Islander, Black, White, or other and used a separate category for Hispanic or Latino or not Hispanic or Latino ethnicity. Race and ethnicity were included to describe the cohorts.

This retrospective analysis included participants who did not follow commands on the day of acute hospital admission and survived to discharge (eMethods in Supplement 1). The primary outcome was a 1-year Glasgow Outcome Scale Extended (GOSE) score less than 4, indicating death or dependency.

Trained study staff followed standard operating procedures to review electronic health records and identify the date of command-following (eMethods in Supplement 1). Among participants who followed commands during acute care, we fit a logistic regression model in each cohort to quantify the association between time to command-following and outcomes. A third model tested for an interaction between cohort and time to command-following. In each cohort, we used linear models to estimate the increase in the proportion of participants with 1-year death or dependency for each additional day without command-following. *P* values were 2-sided, and statistical significance was set at *P* < .05. Data were assessed using R version 4.3.1 (R Project for Statistical Computing) from January 2023 to August 2024.

## Results

This analysis included 9052 participants (mean [SD] age, 38 [18] years; 6841 [76%] male) from the TBIMS cohort and 228 participants (mean [SD] age, 37 [16] years; 174 [76%] male) from the BTRC cohort. Cohorts differed by TBI severity, the proportion receiving inpatient rehabilitation, and 1-year GOSE (Table). Among participants who followed commands during acute care (Figure, A and B), each additional week without command-following was associated with increased odds of death or dependency at 1 year, and the finding was consistent across cohorts (TBIMS: odds ratio [OR], 1.3 [95% CI, 1.3-1.4]; *P* < .001; area under the receiver operating curve [AUC], 0.6; BTRC: OR, 1.5 [95% CI, 1.2-2.0]; *P* = .003; AUC, 0.6; combined model: cohort × command-following interaction, 0.9 [95% CI, 0.7-1.2]; *P* = .41).

**Table.** Cohort Characteristics

Characteristic	Participants, No. (%)		*P* value
	TBIMS (n = 9052)	BTRC (n = 228)	
Age, mean (SD), y	38 (18)	37 (16)	.30
Sex			
Male	6841 (76)	174 (76)	.86
Female	2208 (24)	54 (24)	
Missing	3 (<1)	0	
Race^a^			
Asian or Pacific islander	224 (2)	3 (1)	<.001
Black	1554 (17)	16 (7)	
Hispanic	1075 (12)	NA	
White	6040 (67)	207 (91)	
Other	151 (2)	2 (1)	
Missing	8 (<1)	0	
Ethnicity^b^			
Hispanic or Latino	NA	2 (1)	<.001
Not Hispanic or Latino	NA	192 (84)	
Missing	NA	34 (15)	
Marital status			
Single	4636 (51)	130 (57)	<.001
Married	2820 (31)	55 (24)	
Other	1592 (18)	10 (4)	
Missing	4 (<1)	33 (14)	
Injury mechanism			
High-velocity	5169 (57)	166 (73)	<.001
Fall-related	1987 (22)	47 (21)	
Low-velocity or other	1895 (21)	14 (6)	
Missing	1 (<1)	1 (<1)	
Injury year, median (IQR)	2010 (2006-2015)	2010 (2008 2013)	.85
ED GCS_Total_			
Median (IQR)^c^	10 (4-13)	6 (5-7)	<.001
Missing	5920 (65)	0	
Craniectomy^d^			
Yes	1986 (22)	106 (46)	<.001
No	6924 (76)	122 (54)	
Missing	142 (2)	0	
SDH or SAH			
Present	7217 (80)	186 (82)	.01
Absent	1680 (18)	25 (11)	
Missing	155 (2)	17 (7)	
EDH			
Present	1127 (12)	38 (17)	<.001
Absent	7768 (86)	85 (37)	
Missing	157 (2)	105 (46)	
IVH			
Present	2737(30)	41 (18)	.87
Absent	6161 (68)	88 (39)	
Missing	154 (2)	99 (43)	
Contusions			
Present	6249 (69)	91 (40)	.31
Absent	2645 (29)	47 (21)	
Missing	158 (2)	90 (39)	
Followed commands	8141 (90)	144 (63)	<.001
Time to command-following, Median (IQR), d	5 (2-12)	10 (3-18)	<.001
Acute care LOS, mean (SD), d	25 (19)	27 (15)	.01
Attended acute rehabilitation			
Yes	9052 (100)	159 (70)	<.001
Missing	0	0	
GOSE <4	2112 (23)	96 (42)	<.001
Died	275 (3)	29 (13)	<.001

Abbreviations: BTRC, Brain Trauma Research Center; ED, emergency department; EDH, epidural hematoma; GCS, Glasgow Coma Scale; GOSE, Glasgow Outcome Scale Extended; IVH, intraventricular hemorrhage; LOS, length of stay; NA, not applicable; SAH, subarachnoid hemorrhage; SDH, subdural hematoma; TBIMS, TBI Model Systems.

^a^ By patient or caregiver report. Other race includes individuals who did not self-report as any of the provided groups. BTRC did not collect Hispanic or Latino ethnicity as a racial category but as a separate ethnicity category.

^b^ By patient or caregiver report; TBIMS collected Hispanic or Latino as a racial category; ethnicity was only collected as independent from race starting in 2012.

^c^ In TBIMS, GCS was considered unscorable if a patient was intubated, sedated, or paralyzed.

^d^ TBIMS began collecting this variable January 1, 2007.

**Figure.**
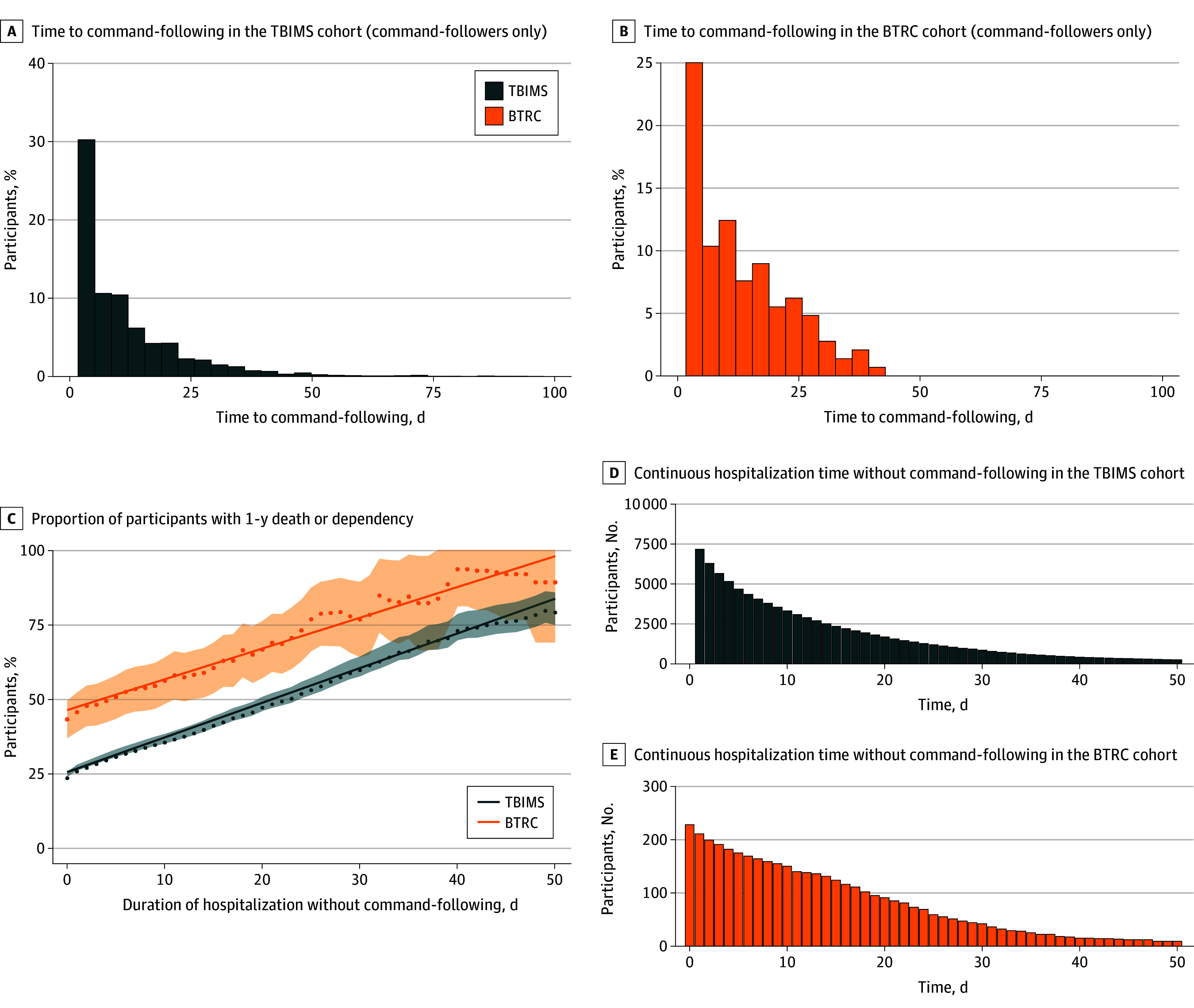
Command-Following and 1-Year Outcomes One-year death or dependency was defined as Glasgow Outcome Scale Extended less than 4. The solid line represents the line of best fit of the individual data points (dots); shading 95% CIs of the daily proportions calculated using the formula for the standard error of a proportion. BTRC indicates Brain Trauma Research Center; TBIMS, TBI Model Systems.

Among all participants, each additional day without command-following was associated with a 1.2% (95% CI, 1.2-1.2) (*P* < .001) increase in TBIMS and 1.1% (95% CI, 1.0-1.1) (*P* < .001) increase in BTRC in the proportion with death or dependency (Figure, C). A 90% or greater likelihood of this outcome was only observed in participants who did not follow commands for at least 50 days in the TBIMS cohort or at least 40 days in the BTRC cohort (Figure, D and E).

## Discussion

In 2 TBI cohorts, time to command-following discriminated weakly between 1-year outcomes. Each additional day without command-following yielded only an approximately 1% increase in probability of death or dependency, a rate remarkably consistent between cohorts. A 90% likelihood of this outcome was observed only in participants failing to follow commands for more than 40 days, a rare occurrence in either cohort, even among those with 1-year death or dependency. These findings suggest that clinicians should avoid confidently assigning a poor prognosis based on the failure to follow commands within the first 5 weeks after TBI.

Limitations of this study include retrospectively determined command-following dates, variability in the number of command-following assessments across participants, and unmeasured confounders associated with critical illness. Additionally, to avoid self-fulfilling prophecy bias, we removed participants who died during acute hospitalization, which may have systematically excluded those more likely to remain dependent. When establishing a prognosis after severe TBI, clinicians may consider applying the 1% rule, that is, the probability of an unfavorable outcome increases by approximately 1% for each additional day without command-following.

## Notes

Supplement 1.
eMethods.

Supplement 2.
Data Sharing Statement
