# Supplementary material for: Time to Command-Following and Outcomes After Traumatic Brain Injury
Source: JAMA Netw Open. 2024 Dec 10;7(12):e2449928. doi: 10.1001/jamanetworkopen.2024.49928 (PMC11632539; doi:10.1001/jamanetworkopen.2024.49928)
Supplement: Supplement 1. — eMethods. [file jamanetwopen-e2449928-s001.pdf]

Supplemental Online Content

Snider SB, Deng H, Hammond FM, et al. Time to command-following and outcomes after traumatic brain injury. *JAMA Netw Open*. 2024;7(12):e2449928. doi:10.1001/jamanetworkopen.2024.49928

eMethods

This supplemental material has been provided by the authors to give readers additional information about their work.

© 2024 Snider SB et al. *JAMA Network Open.*

Supplementary Methods

Command-Following: Definition and Analysis

Trained study staff followed standard operating procedures to review medical charts and identify the date of command-following. In TBIMS chart review was performed by a trained research assistant (RA) who was certified, and biannually re-certified for medical record abstraction, following satisfactory completion of an online examination. This test includes abstracting information (including date of command-following) from a standard, anonymized online medical record. Each study center is required to have an independent and certified staff member re-abstract all variables from one participant's record each quarter. If *any* errors are identified, they are corrected, and an additional case is re-abstracted with this same procedure repeated. BTRC command-following dates were extracted from the medical chart by either an MD neurosurgeon or trained RA.

TBIMS standard operating procedures define command-following as the first occurrence of command-following documented in any two clinical notes within 24 hours. If the notes fell on consecutive hospital days, the second day was used as the date of command-following. In BTRC, command-following was defined as the first occurrence of command-following documented in a physician note on two consecutive hospital days. The observation period for command-following spanned from acute hospital admission to discharge.

Among participants who followed commands during the observation window (8,141 [90%] participants in TBIMS; and 144 [63%] in BTRC), we used logistic regression and area under the receiver operating characteristic curve (AUC) to quantify the association between weeks to command-following and 1-year death or dependency. We initially fit separate univariate models to obtain a crude (unadjusted) odds ratio for each dataset. To specifically test the hypothesis that the association between time to command-following and outcome was the same across

© 2024 Snider SB et al. *JAMA Network Open.*

datasets, we fit a third model with predictors including: cohort [TBIMS vs BTRC], time to command-following, and the interaction between cohort x time to command following. We report the p value for the coefficient of this interaction term in the main text.

The intention of this study was to quantify the association between time to command-following and outcomes, and measure its consistency across TBI studies, despite known heterogeneity in clinical contexts. Thus, we report only crude odds ratios, rather than a full prediction model, which would require inclusion of covariates known to be associated with outcome. To identify differences in cohort characteristics, we used T tests for continuous variables and Chi-squared tests for categorical variables.

Absence of Command-following: Definition and Analysis

We computed the proportion of participants with a 1-year outcome of death or dependency among all participants who did not follow commands on or before each of the first 50 days following acute hospital admission. Participants were included in this analysis until they followed commands or were discharged from the acute hospital. 50 days was chosen as the endpoint, as beyond 50 days, there were fewer than 10 participants remaining in the BTRC cohort. In each cohort, we used linear models to estimate the increase in the proportion of participants with 1-year death or dependency for each additional day without command-following.

© 2024 Snider SB et al. *JAMA Network Open.*

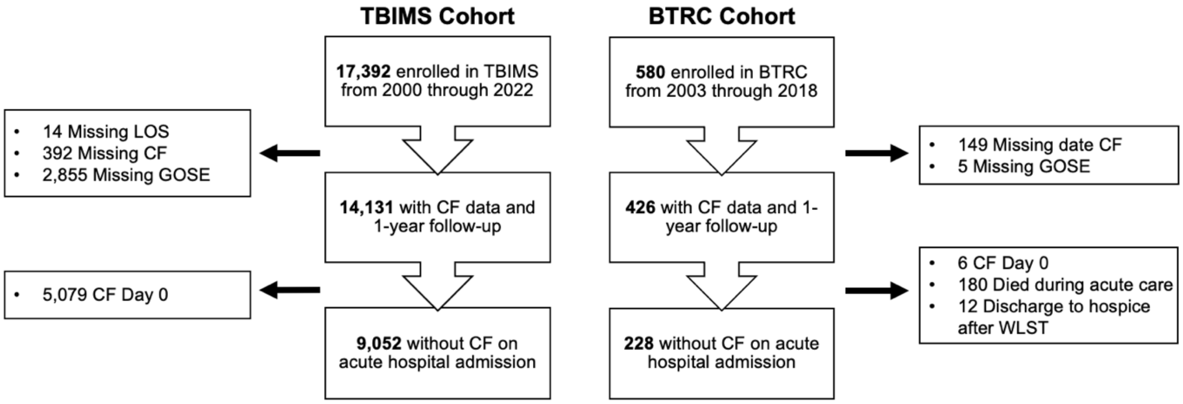

Supplementary Figure 1: Study Flowchart
Study CONSORT diagram. Abbreviations: CF = command-following; LOS = length of stay; GOSE = Glasgow Outcome Scale Extended; WLST = withdrawal of life sustaining treatment

© 2024 Snider SB et al. *JAMA Network Open.*
