# Supplementary material for: Time to Command-Following and Outcomes After Traumatic Brain Injury
Source: JAMA Netw Open. 2024 Dec 10;7(12):e2449928. doi: 10.1001/jamanetworkopen.2024.49928 (PMC11632539; doi:10.1001/jamanetworkopen.2024.49928)
Supplement: Supplement 2. — Data Sharing Statement [file jamanetwopen-e2449928-s002.pdf]

Data Sharing Statement

Data
Data available: No

Additional Information
Explanation for why data not available: It will be made available pending proposal to the PIs of the two studies (TBI-MS and BTRC)
